# Prognostic significance of CD44s expression in resected non-small cell lung cancer

**DOI:** 10.1186/1471-2407-11-340

**Published:** 2011-08-07

**Authors:** Yoon Ho Ko, Hye Sung Won, Eun Kyoung Jeon, Sook Hee Hong, Sang Young Roh, Young Seon Hong, Jae Ho Byun, Chan-Kwon Jung, Jin Hyoung Kang

**Affiliations:** 1 Division of Oncology, Department of Internal Medicine, Uijeongbu St. Mary's Hospital, Catholic University, Uijeongbu -si, Gyeonggi-do, South Korea; 2 Division of Oncology, Department of Internal Medicine, Seoul St. Mary's Hospital, Catholic University, Seoul, South Korea; 3 Division of Oncology, Department of Internal Medicine, Incheon St. Mary's Hospital, Catholic University, Seoul, South Korea; 4 Department of Hospital Pathology, Seoul St. Mary's hospital, Catholic University, Seoul, South Korea

**Keywords:** non-small cell lung cancer, CD44s, immunohistochemistry, prognosis

## Abstract

**Background:**

CD44s is a cell adhesion molecule known to mediate cellular adhesion to the extracellular matrix, a prerequisite for tumor cell migration. CD44s plays an important role in invasion and metastasis of various cancers. In the present study, we sought to determine whether CD44s is involved in clinical outcomes of patients with resected non-small cell lung cancer (NSCLC).

**Methods:**

Using immunohistochemical staining, we investigated CD44s protein expression using tissue array specimens from 159 patients with resected NSCLC (adenocarcinoma (AC; *n *= 82) and squamous cell carcinoma (SCC; *n *= 77). Additionally, the immunoreactivity of cyclooxygenase (COX)-2 was also studied. The clinicopathological implications of these molecules were analyzed statistically.

**Results:**

High CD44s expression was detected more frequently in NSCLC patients with SCC (66/72; 91.7%) than in those with AC histology (*P <*0.001). Additionally, high CD44s expression was significant correlated with more advanced regional lymph node metastasis (*P *= 0.021). In multivariate analysis of survival in NSCLC patients with AC histology, significant predictors were lymph node metastasis status (*P *< 0.001), high-grade tumor differentiation (*P = *0.046), and high CD44s expression (*P = *0.014). For NSCLC patients with SCC histology, the significant predictor was a more advanced tumor stage (*P = *0.015). No significant association was found between CD44s and clinical outcome (*P *= 0.311).

**Conclusions:**

High CD44s expression was a negative prognostic marker with significance in patients with resected NSCLC, particularly those with AC histology, and was independent of tumor stage.

## Background

Cell adhesion molecules control cell behavior through interactions with each other and with their microenvironment by exchanging information through cell-cell and cell-extracellular matrix (ECM) interactions [1]. As a cell adhesion molecule, CD44, a widely expressed cell-surface glycoprotein and a cell-surface receptor for hyaluronate and osteopontin, is known to mediate cellular adhesion to the ECM, which is a prerequisite for tumor cell migration [2]. CD44 is the product of a single gene located on the short arm of human chromosome 11; the entire human CD44 gene is encoded by 60 kb of genomic DNA and contains 20 exons [3]. Its low molecular weight standard isoform, CD44s, binds more strongly to hyaluronate than high molecular weight CD44 isoforms. The intracellular domain of CD44s interacts with the cell cytoskeleton by binding directly to ankyrin, and the interaction between ankyrin and CD44s is required for the modulation of CD44s cell surface expression and adhesion functions. CD44 has been reported to play an important role in cancer cell invasion and metastasis as well as in fundamental biological processes, including lymphocyte homing, hematopoiesis, inflammation, wound healing, and apoptosis [4,5].

The expression pattern of CD44 showed contradictory results in various cancer types. CD44 expression is up-regulated in several tumor types, such as breast cancer [6], gastric cancer [7], bladder cancer [8], head and neck cancer [9], colon cancer [10], hepatocellular carcinoma [4]. The down-regulation of CD44 has been reported in other cancer types, including carcinoma arising from the progression to dysplasia in Barrett's esophageal epithelium [11], metastatic endometrial cancer [12], high-grade bladder transitional cell carcinoma [13], prostate cancer [14], and high-grade brain tumors [15]. Considerable variability exists in the clinical implications of CD44s in various cancers. Some studies have demonstrated that CD44 overexpression is related to more aggressive disease [4,6-10], while others have shown that loss of CD44 expression is associated with poor prognosis [11-15]. Similar conflicts are observed in reports regarding lung cancer [16-20]. The activation of CD44 isoforms also triggers resistance to chemotherapeutic agents in non-small cell lung cancer (NSCLC) and colon cancer cell lines [21,22].

Cyclooxygenase (COX) is the key enzyme in prostaglandin metabolism. COX-2 has been reported to be highly expressed in various malignancies. COX-2 expression has been suggested to be correlated with tumor aggressiveness and poor prognosis. Earlier studies have observed individual roles for CD44 and COX in embryogenesis, angiogenesis, cellular proliferation, wound healing, as well as in pathophysiological conditions, such as cancer and inflammation. COX-2 overexpression is known to be as a proximal mediator of CD44-dependent invasion in NSCLC cells [23].

Thus, we investigated histogenic differences of the immunostaining pattern of CD44s in surgical specimens from NSCLC patients and their correlation with clinicopathological parameters and patient clinical outcomes. We also investigated COX-2 expression to determine any association with CD44s, clinicopathological parameters, and clinical outcome in these patients.

## Methods

### 1. Patients and tissue specimens

All tissues investigated in the present study were obtained from consecutive NSCLC patients. We examined two histological tumor subtypes, adenocarcinoma (AC) and squamous cell carcinoma (SCC), to compare the role of investigated markers based on histological type. In total, 159 consecutive patients underwent curative (R0) resection (lobectomy or pneumonectomy) between April 1997 and March 2003 at Seoul St. Mary's Hospital, which is affiliated with Catholic University of Korea. The research was approved by the hospital's Institutional Review Board. Patient clinicopathological data obtained from medical records included age, gender, histopathological diagnosis, pathological tumor stage, and the date of initial diagnosis, relapse, death, or the last follow-up. Histological classification was performed according to the World Health Organization (WHO) criteria and post-operative pathological staging was performed according to the American Joint Committee on Cancer (AJCC) staging criteria, 6^th ^edition.

### 2. Construction of the tissue microarray

All archival tissue samples were routinely fixed in formalin and embedded in paraffin wax. Representative tissue areas were marked on standard hematoxylin and eosin (HE) sections, punched out of the paraffin block using a 2.0-mm punch, and inserted into a recipient paraffin block. To decrease error introduced by sampling and to minimize the impact of tissue loss during processing, duplicate tissue cores per specimen were arrayed on a recipient paraffin block. Sections (4 μm) were cut from the completed array block and transferred to silanized glass slides.

### 3. Immunohistochemistry and analysis

Tissue sections were deparaffined by incubation in xylene and rehydrated through graded ethanol-water solutions. Endogenous peroxidase was blocked with 3% H_2_O_2 _in methanol. Antigen retrieval was performed with citrate buffer (pH 6.0) by heating in a microwave vacuum histoprocessor (RHS-1; Milestone, Bergamo, Italy) at a controlled final temperature of 121°C for 15 min, and then cooling to room temperature for 15 min.

The CD44s polyclonal antibody (R&D Systems, Minneapolis, MN) was diluted to 1:10 using a dilution buffer. The COX-2 monoclonal antibody (Cayman, Ann Arbor, MI) was diluted to 1:100. The primary antibodies were incubated at room temperature for 1 h. Detection was performed using a conventional labeled streptavidin/biotin method (LSAB2 System-HRP; Dako). The color reaction was incubated in 3,3'diaminobenzidine for 5 min and hematoxylin counterstaining was used. CD44s and COX-2 immunostains were interpreted by a pathologist (C.K.J.) who was blinded to the specific diagnosis and prognosis of each case. For CD44s staining, staining intensities were scored as no staining (0), weak staining (1+), moderate staining (2+), or strong staining (3+). The percentage of staining area was classified as follows: 0, 0%; 1, 1-10%; 2, 11-50%; 3, 51-100%. The intensity and percentage scores were multiplied to yield a composite score of 1 to 9 for each specimen. Composite scores of 1 3 were defined as indicating low CD44s protein expression, and scores of 4 9 were considered to indicate high CD44s expression. Additionally, for CD44s, only cell membranous staining was considered to be positive. For COX-2 staining, tumors were considered to demonstrate positive expression if ≥ 10% of tumor cells were immunostained.

### 4. Statistical analysis

Statistical analyses were performed using the SPSS software (ver. 13.0; SPSS, Chicago, IL). Survival was determined from the date of surgery to the time of an event (recurrence or death) using the Kaplan-Meier method. Following an intent-to-treat approach, non-cancer-related deaths as well as follow-up loss were included in survival analyses, but were considered to be censored data. Relationships among CD44s expression, COX-2 expression, and clinicopathological features were evaluated using Spearman's rank coefficient or Fisher's exact probability test. The statistical significance of differences in cumulative survival curves was evaluated using the log-rank test. Multivariate survival analysis was performed on all parameters that were found to be significant in univariate analyses using the Cox proportional hazards model by histological stratification. Survival rates and odds ratios are presented with their 95% confidence intervals (CIs). Statistical tests were two-sided at the 5% level of significance.

## Results

### 1. Patient clinical characteristics

In total, 159 paraffin blocks with tumor samples were available from patients who had undergone surgery. Regarding treatment, no patient received radiation or chemotherapy preoperatively. The median follow-up duration was 49.9 months (range, 0.6-148.3 months) after the initial pathological diagnosis. Among the 159 patients, 64 (40.3%) died of their tumors and 95 (59.7%) were still alive at the last follow-up. The overall 5-year survival rate for resected NSCLC was 70.4%. The clinical and pathological characteristics of the series are shown in Table 1. The patients consisted of 123 males and 36 females, with a median age of 64 years (range, 19-88 years). Histologically, 82 patients (51.6%) had AC and 77 (48.4%) had SCC. Of those 159 patients, 26 (16.4%) had well differentiated, 96 (60.4%) had moderately differentiated, and 37 (23.3%) had poorly differentiated carcinoma. In total, 77 (48.4%) patients received adjuvant treatment. Patient characteristics were similar between SCC and AC patients, but advanced age (*P *= 0.024) and a male predominance (*P *< 0.001) were observed in SCC patients. Well-differentiated tumors were observed more commonly in AC patients.

**Table 1:** Clinical and pathologic characteristics of patients (*n *= 159)

Characteristics	AC		SCC		
	
	No. of patients	%	No. of patients	%	p-value
No. of patients included	82		77		
Age (years), median (range)	62.0 (19-82)		64.0(33-88)		
< 60	35	42.7	22	28.6	**0.024**
≥ 60	47	57.3	55	71.4	
Sex					
Male	51	63.4	72	93.5	**< 0.001**
Female	31	37.8	5	6.5	
Tumor size (n = 157)					
< 3 cm	29	35.8	20	26.3	0.061
≥ 3 cm	52	64.2	56	73.7	
T (n = 158)					
1-2	72	87.8	61	80.3	0.076
3-4	10	12.8	15	19.7	
Lymph node metastasis					
Negative	49	59.8	47	61.0	0.127
Positive	33	40.2	30	39.0	
Stage					
I	43	50.6	42	57.8	0.072
II	18	18.1	17	20.0	
III	21	20.1	18	22.2	
Tumor differentiation					
Well	22	26.8	4	5.2	**0.031**
Moderately	41	50.0	55	71.4	
Poorly	19	23.2	18	23.4	
Adjuvant treatment					
not done	45	54.9	37	45.1	0.241
done	37	45.1	40	51.9	
CD44s (n = 149)					
Low	36	46.8	6	8.3	**< 0.001**
High	41	53.2	66	91.7	
COX-2 (n = 159)					
Low	16	19.5	23	29.9	0.129
High	66	80.5	54	70.1	

AC, adenocarcinoma; SCC, squamous cell carcinoma, COX, cyclooxygenase

### 2. Immunohistochemical staining patterns

**Figure 1:**
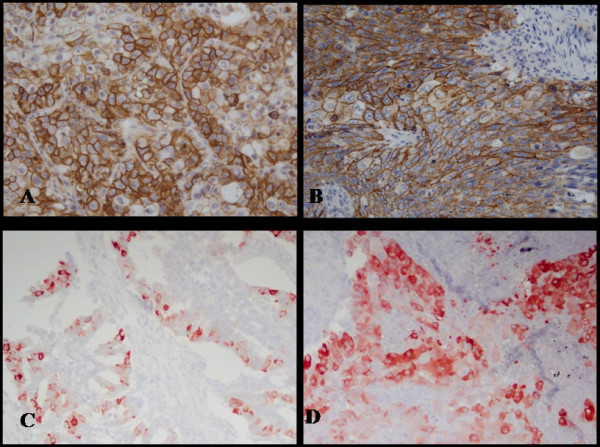
**Microscopic views of positively stained immunohistochemistry sections in non-small cell lung cancer**. Immunoreactivity for CD44s was detected predominantly in the membrane of tumor cells. Typical examples of CD44s immunopositivity in AC (×400; A) and SCC (×400; B) histological subtypes. Immunoreactivity of COX-2 was detected predominantly in the cytoplasm of tumor cells. COX-2 immunopositivity in AC (×400; C) and SCC (×400; D) histological subtypes.

Of 159 consecutive patients, we were unable to analyze, 10 (6.3%, AC 5, SCC 5) of the punches for CD44s, due to tissue loss during processing. Of 149 specimens, CD44s-positive tumors, which were predominantly observed in the membrane of the cancer cells (Figure 1A, B), were demonstrated in 107 (67.3%) and were found more frequently with the SCC histological subtype (91.7%; *P <*0.001) compared with the AC histological subtype (Table 1). Of 159 specimens, COX-2 was highly expressed in the cytoplasm of tumor cells (Figure 1C, D) with a positive rate of 75.5%. No significant difference in COX-2 expression was observed between AC and SCC patients.

### 3. Correlation between expression of investigated factors and clinicopathological features in AC and SCC cases

In Table 2, associations among CD44s expression, COX-2 expression, and clinicopathological features in AC cases are shown. High CD44s expression was significantly correlated with more advanced regional lymph node metastasis (*P *= 0.021). High COX-2 expression was significantly correlated with small tumors (*P *= 0.009), but was not significantly related to lymph node metastasis. However, increased expression of CD44s and COX-2 was not significantly related to any clinicopathological feature in SCC patients (Table 3). We also explored the association between CD44s and COX-2 expression. However, no significant correlation was found among the investigated markers in AC and SCC patients (Table 4).

**Table 2:** Clinicopathological factors and their relationship to the expression of different proteins, assessed in AC cases

Characteristics	CD44s (n = 77)		COX-2 (n = 82)	
	
	Low(%)	High(%)	Low(%)	High(%)
Tumor differentiation				
Well,	7(33.3)	14(66.7)	7(31.8)	15(68.2)
Moderately, poorly	29(51.8)	27(48.2)	9(15.0)	51(85.0)
p-value		0.117		0.085
Tumor size				
< 3 cm	12(42.9)	16(57.1)	1(3.4)	28(96.6)
≥ 3 cm	24(50.0)	24(50.0)	14(26.9)	38(73.1)
p-value		0.547		**0.009**
Lymph node metastasis				
N0-1	33(53.2)	29(46.8)	14(20.9)	539(79.1)
N2-3	3(20.0)	12(80.0)	2(13.3)	13(86.7)
p-value		**0.021**		0.397
Stage				
I	20(48.8)	21(51.2)	10(23.3)	33(76.7)
II	8(53.3)	7(46.7)	3(16.7)	15(83.3)
III	8(38.1)	13(61.9)	3(14.3)	18(85.7)
p-value		0.616		0.373
Lymphatic invasion				
Negative	19(42.2)	26(57.8)	11(23.4)	36(76.6)
Positive	16(53.3)	14(46.7)	5(15.2)	28(84.8)
p-value		0.345		0.364

AC, adenocarcinoma; COX, cyclooxygenase.

**Table 3:** Clinicopathological factors and their relationship to the expression of different proteins, assessed in SCC cases

Characteristics	CD44s (n = 72)		COX-2 (n = 77)	
	
	Low(%)	High(%)	Low(%)	High(%)
Tumor differentiation				
Well	0(0)	4(100)	1(25.0)	3(75.0)
Moderately, poorly	6(8.8)	62(91.2)	22(30.1)	51(69.9)
p-value		0.701		0.655
Tumor size				
≤ 3 cm	1(5.6)	17(94.4)	6(30.0)	14(70.0)
> 3 cm	4(7.5)	497(92.5)	20(37.7)	33(62.3)
p-value		0.625		0.976
Lymph node metastasis				
N0-1	5(8.1)	57(91.9)	21(31.3)	46(68.7)
N2-3	1(10.0)	9(90.0)	2(20.0)	8(80.0)
p-value		0.607		0.373
Stage				
I	3(7.9)	35(92.1)	12(28.6)	30(71.4)
II	1(6.3)	15(93.8)	4(23.5)	13(76.5)
III	2(11.1)	16(88.9)	7(38.9)	11(61.1)
p-value		0.736		0.594
Lymphatic invasion				
negative	5(13.2)	33(86.8)	11(27.5)	29(72.5)
positive	1(3.3)	29(96.7)	11(33.3)	22(66.7)
p-value		0.163		0.589

SCC, squamous cell carcinoma; COX, cyclooxygenase.

**Table 4:** Relationship between the expression patterns of markers according to histological subtype

		**AC**		**SCC**	
			
		**COX-2**		**COX-2**	
			
		**Low(%)**	**High(%)**	**Low(%)**	**High(%)**
	
CD44s	Low(%)	9(25.0)	27(75.0)	1(16.7)	5(83.3)
	High(%)	6(14.6)	35(85.4)	21(31.8)	451(68.2)
	p-value	0.252			0.400

AC, adenocarcinoma; SCC, squamous cell carcinoma; COX, cyclooxygenase.

### 4. Overall survival with respect to clinicopathological factors

**Figure 2:**
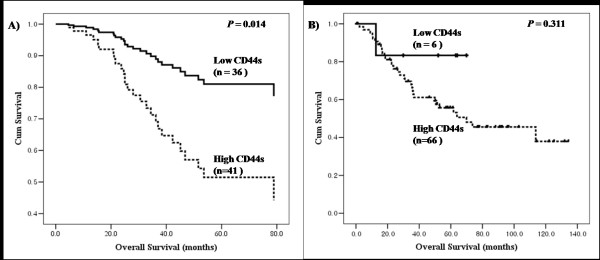
**Overall survival of non-small cell lung cancer patients were stratified by CD44s expression according to histological subtype**. A) Overall survival for 82 adenocarcinoma patients. B) Overall survival for 77 squamous cell carcinoma patients.

For AC, univariate analysis of clinicopathological factors relevant to patient survival revealed that the following factors were statistically significant for the overall survival of patients: regional lymph node metastasis status (*P <*0.001), advanced tumor node metastasis (TNM) stage (*P <*0.001), lymphatic invasion status (*P <*0.001), advanced age (*P *= 0.027), and poor tumor differentiation (*P *= 0.046). Additionally, high CD44s expression demonstrated statistical significance for the overall survival of patients (*P = *0.016; data not shown). In multivariate analysis, significant predictors were lymph node metastasis status (hazard ratio, 5.212; 95% CI, 2.222-12.226; *P *< 0.001), high-grade tumor differentiation (hazard ratio, 3.110; 95% CI, 1.020-9.484; *P = *0.046), and high CD44s expression (hazard ratio, 3.152; 95% CI, 1.256-7.910; *P = *0.014; Table 5, Figure 2). For SCC, univariate analysis of clinicopathological factors relevant to patient survival revealed that the following factors were statistically significant for the overall survival: more advanced T stage (*P <*0.001), advanced TNM stage (*P <*0.001), regional lymph node metastasis status (*P *= 0.004), and lymphatic invasion status (*P *= 0.039). However, no association was observed with the investigated markers (data not shown). In multivariate analysis for overall survival, the significant predictor was more advanced T stage (hazard ratio, 2.537; 95% CI, 1.202-5.355; *P = *0.015). Lymph node metastasis status was marginally associated with survival (hazard ratio, 2.001; 95% CI, 0.954-4.197; *P = *0.066). No significant association was observed between CD44s expression and clinical outcome (*P *= 0.311; Table 5, Figure 2). We also analyzed prognostic factors using multivariate analysis for the overall population. Lymph node metastasis status (hazard ratio, 3.553; 95% CI, 1.826-5.502; *P *< 0.001) and advanced T stage (hazard ratio, 1.826; 95% CI, 0.996-3.349; *P *= 0.052) were strong predictors for survival.

**Table 5:** Multivariate analysis of clinicopathological characteristics and biological factors by overall survival rate

Characteristics	Hazards ratio	95% CI	*P*-value
Total cases			
Lymph node metastasis (no/yes)	3.553	1.826-5.502	**< 0.001**
T stage (T1-2/T3-4)	1.826	0.996-3.349	0.052

AC			
Lymph node metastasis (no/yes)	5.212	2.222-12.226	**< 0.001**
CD44s (low/high)	3.152	1.256-7.910	**0.014**
Tumor differentiation (well/moderately, poorly)	3.110	1.020-9.484	**0.046**

SCC			
T stage (T1-2/T3-4)	2.537	1.202-5.355	**0.015**
Lymph node metastasis (no/yes)	2.001	0.954-4.197	0.066
CD44s (low/high)			0.311

CI, confidence interval; AC, adenocarcinoma; SCC, squamous cell carcinoma.

## Discussion

The present study demonstrated that the staining patterns of CD44s in NSCLC tissue varied according to histological subtype. Additionally, high CD44s expression was a negative prognostic marker with significance in patients with resected NSCLC, particularly those with AC histology, and was independent of tumor stage.

In the present study, the frequency of CD44s expression in all cases was 67.3% and this was observed more frequently with SCC (91.7%) than in AC (53.2%) cases. This tendency was observed in previous reports [18,24,25]. A recent report also showed that high expression of CD44v6, a CD44 isoform, was detected more frequently in SCC than in other carcinoma types [16]. Squamous cell carcinoma derives from dysplastic or metaplastic stratified epithelia (i.e., from bland squamous metaplasia to squamous cell carcinoma in situ, and then finally to invasive carcinoma). In a recent study by Leung et al., evaluating non-cancerous lung tissue, membrane expression of CD44 was confined to the surface of bronchial basal cells, alveolar macrophages, and regenerating cuboidal pneumocytes of injured lung, whereas no CD44 expression was observed in terminally differentiated epithelial cells such as ciliated or non-ciliated columnar cells of bronchial epithelium or type I flat pneumocytes lining alveolar spaces [18]. In contrast, squamous metaplasia showed strong CD44 immunoreactivity in the proliferating basal layers, while in premalignant dysplasia, the entire thickness displayed aberrant CD44 expression, indicating that squamous malignant transformation is closely associated with CD44 expression. Thus, squamous cell carcinoma essentially recapitulates the staining pattern of normal bronchial basal cells. However, adenocarcinoma of the lung is usually accompanied by a morphological decrease in and subsequent loss of CD44 [16-18]. This discrepancy may be related to the heterogeneous histogenetic origin of adenocarcinoma of the lung. Additionally, the functional role of CD44 has revealed different features of the molecule in the two histological subtypes. In SCC, only cells of the neoplastic stage can transduce subcellular signals via CD44 [26], whereas CD44s down-regulation in the NSCLC cell line H322, derived from a primary bronchioalveolar carcinoma of the lung, may confer a protective advantage by allowing escape from tumoricidal effector cells, including activated macrophages of the host, thus promoting tumorigenesis [27].

The exact role of CD44s and its various isoforms in tumorigenesis and tumor progression remains controversial. For example, Liu et al. considered that the expression of CD44s in gastric cancer tissue was much higher than that in normal tissue and its high expression was associated with tumorigenesis, metastasis, and the clinically aggressive behavior of the adenocarcinoma [28]. However, loss of CD44s expression has been found to correlate with lymph node metastasis and unfavorable outcome in patients with breast carcinomas [29]. The findings of the present study, strong association between tumor tissue expression of CD44s and advanced lymph node metastasis in the AC histological subtype (*P *= 0.021), are also controversial in the literature regarding NSCLC. According to the report by Tran et al., concerning the analysis of formalin-fixed, paraffin-embedded archival tissues from 49 SCC and 49 AC cases, CD44s expression did not correlate with tumor stage, recurrence, and survival rates, whereas expression of the variant isoform, CD44v6, was particularly associated with local lymph node metastasis in NSCLC [17]. Miyoshi et al. also obtained a similar result, that increased CD44v6 expression was associated with lymph node metastasis in NSCLC [19]. However, considering only NSCLC with AC histology, the level of CD44v6 expression had no significant association with lymph node metastases or tumor stage in the study by Ramasami et al. [20] Thus, it seems reasonable to predict that tumor invasion and metastasis could be enhanced by the cell-ECM interaction in CD44-overexpressed tissue or by cell-cell interaction in CD44-deficient tissue.

Large-scale studies have shown that tumor stage is the most consistent prognostic factor in patients with NSCLC [30]. This fact accords with our current study in which T and N stages (*P *< 0.001 and *P *= 0.052, respectively) were strong prognostic factors for overall survival. We also explored the value of CD44s as a molecular prognostic indicator according to NSCLC histology and found that high CD44s expression predicted a poor post-operative survival for curatively resected NSCLC patients with AC histology (*P *= 0.014). For several other cancers, while several studies have shown that overexpression of CD44s and its splice variants, particularly CD44v6, are associated with poor prognosis and metastasis [4,6-10], others have shown that downregulation of CD44 expression correlated with adverse outcome [11-15]. The exact role of CD44 in the biological behavior and clinical prognosis of NSCLC also remains to be defined. Hirata et al. reported that the 5-year survival rate of patients with CD44v6-positive tumors was 50%, which was significantly lower than that of CD44v6-negative patients (88%; *P *= 0.001), in 69 patients with stage I NSCLC, 43 of whom had AC, and suggested that CD44v6 expression could predict poor prognosis [31]. In contrast, recent reports have shown that patients with tumors exhibiting low-level CD44 or CD44v6 expression had a worse post-operative survival than those with high levels of CD44 in resected NSCLC patients with AC or SCC histology [16,18]. Also, in Tran et al.'s report, CD44s expression had no prognostic role in the analysis of 49 SCC and 49 AC tissue specimens [17]. These differing results in NSCLC may be partially attributed to differences in antibodies used for staining, different criteria for defining stain positivity, and different patient demographics such as histological subtype, tumor stage, adjuvant treatment, and insufficient SCC immunoreactivity. However, obviously, CD44 is a membrane-bound glycoprotein and mediates a complex range of functions. Invasive and metastatic growth can be mediated through the interaction of cell surface CD44 with ECM components such as hyaluronan [32] or cell-cell interactions [1]. CD44-hyaluronan aggregates are essential for activation of metalloproteinase, which induces tissue invasion and tumor growth factor-β [33]. Signaling through CD44 in endothelial cells is also known to induce COX-2 via vascular endothelial growth factor (VEGF) generation, proliferate endothelial cells, and increase neo-vascularization [34]. CD44 expressed on the surface of cancer cells has been shown to facilitate binding to endothelial P- or L-selectin and increase tumor access to hematogenous spread [35]. Additionally, CD44 acts as a co-receptor with neighboring receptor tyrosine kinases, such as epidermal growth factor receptor (EGFR) [36], hepatocyte growth factor receptor (c-MET) [37] and VEGF receptor (VEGFR) [38], and induces the PI3K/AKT cascade and anti-apoptotic pathway [39], resulting in enhanced aggressiveness and multi-drug resistance [40]. Recently, CD44 has also been reported to be a possible cancer stem cell marker for breast, pancreas, or colorectal cancer [41]. In bronchial cells, CD44 is associated with stem cells, namely basal cells and type 2 pneumocytes, and may act to anchor these cells to the matrix and be important in migration during repair or neoplasia. Furthermore, as mentioned above, CD44 expression is maintained throughout tumorigenesis in squamous cell carcinoma and bronchioloalveolar carcinoma, suggesting a histogenetic relationship between stem cells and their respective tumors [18].

Although, in our study, the sample size was not sufficiently large to make a final statement on the prognostic value of CD44, it has clear clinical implications as a therapeutic target. In phase I dose-escalation studies with the humanized IgG1 monoclonal CD44v6 antibody bivatuzumab, labeled with rhenium-186, the radiolabeled antibody showed promising anti-tumor effects with consistent stable disease at higher radioactive dose levels and with only low toxicity in patients with refractory head and neck SCC [42].

## Conclusions

The present study has shown that high CD44s expression may be strongly negatively associated with clinical outcomes in patients with resected NSCLC, particularly for those with AC histology. CD44s expression may be useful in selecting the subgroup with benefit from adjuvant chemotherapy. Although the present study demonstrates clinical implications of CD44, the information provided may suffer from some limitations, such as the retrospective nature of the study, some patient selection bias related to treatment at a single institution, and the identification of a functional role of CD44 in NSCLC that needs to be explored further in a larger cohort of patients and in prospective studies.

## Competing interests

The authors declare that they have no competing interests.

## Authors' contributions

YHK: Study design, statistical analysis, and preparation of the article for publication. CKJ: Implementation of the immunohistochemical procedures, immunohistochemical interpretation, histological examination and grading, immunohistochemical interpretive calibration and peer reviewing the final draft. SYR, HSW, EUK, SHH, MAL, JHK, YSH, JHB: Performing the chemotherapy and management and per reviewing the final draft. All authors read and approved the final manuscript.

## Pre-publication history

The pre-publication history for this paper can be accessed here:

http://www.biomedcentral.com/1471-2407/11/340/prepub
